# Investigation of *ABO* Gene Variants across More Than 60 Pig Breeds and Populations and Other *Suidae* Species Using Whole-Genome Sequencing Datasets

**DOI:** 10.3390/ani14010005

**Published:** 2023-12-19

**Authors:** Matteo Bolner, Francesca Bertolini, Samuele Bovo, Giuseppina Schiavo, Luca Fontanesi

**Affiliations:** Department of Agricultural and Food Sciences, Division of Animal Sciences, University of Bologna, Viale Giuseppe Fanin 46, 40127 Bologna, Italy; matteo.bolner2@unibo.it (M.B.); samuele.bovo@unibo.it (S.B.); giuseppina.schiavo2@unibo.it (G.S.)

**Keywords:** allele, blood type, genomics, polymorphism, *Sus scrofa*

## Abstract

**Simple Summary:**

The *ABO* gene is the major blood classification system, which, in humans, is present in three main forms: *A*; *B*; and *O*. Under the umbrella of these main forms, other variants modulate the activity of the gene for functions that go beyond the determination of the blood type. However, in other animals, this gene is still poorly studied. The pig is a species of economic relevance, an animal model, and one of the most promising species for the development of xenotransplants. For all these reasons, it is important to investigate the *ABO* gene in pigs. In this study, we explored the main alleles present in different pig species (*A* and *O*) and other variants besides these two major forms to investigate the evolution of *ABO* and its possible association with several production and blood-related traits. We reported that the *ABO* gene has multiple variants that helped to define the evolution of the gene across the *Suinae* species. In an explorative analysis, we also reported that *ABO* gene polymorphisms were suggestively associated with the level of magnesium in plasma. Further studies are needed to dissect the effect of *ABO* gene polymorphisms in different pig breeds and populations.

**Abstract:**

Polymorphisms in the human *ABO* gene determine the major blood classification system based on the three well-known forms: *A*; *B*; and *O*. In pigs that carry only two main alleles in this gene (*A* and *O*), we still need to obtain a more comprehensive distribution of variants, which could also impact its function. In this study, we mined more than 500 whole-genome sequencing datasets to obtain information on the *ABO* gene in different *Suidae* species, pig breeds, and populations and provide (i) a comprehensive distribution of the *A* and *O* alleles, (ii) evolutionary relationships of *ABO* gene sequences across *Suidae* species, and (iii) an exploratory evaluation of the effect of the different *ABO* gene variants on production traits and blood-related parameters in Italian Large White pigs. We confirmed that allele *O* is likely under balancing selection, present in all *Sus* species investigated, without being fixed in any of them. We reported a novel structural variant in perfect linkage disequilibrium with allele *O* that made it possible to estimate the evolutionary time window of occurrence of this functional allele. We also identified two single nucleotide polymorphisms that were suggestively associated with plasma magnesium levels in pigs. Other studies can also be constructed over our results to further evaluate the effect of this gene on economically relevant traits and basic biological functions.

## 1. Introduction

The histo-blood group *ABO* system is the major blood human classification system that is defined by the presence in the serum of *A*, *B*, or *O* antigens, expressed as glycoproteins, glycolipids, and antibodies. Such antigens were originally detected in erythrocytes but later found in other predominant epithelial and endothelial cells [1].

The *A*, *B*, and *O* antigens are produced by modifications of the *H* antigen through the glycosyltransferase activity of the *ABO* enzyme. Different alleles of the *ABO* gene determine its enzymatic activity: allele *A* encodes the enzyme α-1,3-*N*-acetylgalactosamine transferase (A-transferase); allele *B* encodes the enzyme α-1,3-galactosyl transferase (B-transferase); and allele *O* lacks enzymatic activity, leaving the *H* antigen without any modification. In humans, four amino-acid substitutions determine the difference between alleles *A* and *B*, while a single base deletion causing a frameshift of the reading frame determines the non-functionality of allele *O*, which results in a protein that is not immunologically related to the A and B transferases [2]. Together with the three main alleles, other polymorphisms have been identified in humans, and so far, hundreds of different secondary alleles have been detected [3,4]. These polymorphisms have been associated with several diseases, e.g., pancreatic cancers, cardiocerebrovascular diseases [5,6], and ischemic stroke [7], and with different hematological parameters [8,9].

The rising interest in xenotransplantation is also driving research on the fine biological roles and functions of the *ABO* gene in other species to reduce the rejection of the donor organ from the human immune system. For example, the expression of porcine α-1,3-galactosyltransferase in human cells has been shown to induce sensitivity to human serum and retroviruses [10].

Orthologs of the human *ABO* gene have been identified in many different mammalian species: Kominato et al. [11] successfully hybridized a human A transferase cDNA probe with several primate species, along with a mouse, chicken, and several other mammal species such as dog, cat, rabbit, cow, sheep, rat, hamster, and marmoset. No hybridization was found with more distantly related organisms, such as bacteria, yeast, nematodes, clams, flies, sea urchins, and frogs.

Additionally, *A*, *B*, and *O* antigens were described in primate species, with the same different amino acids determining the *A* and *B* specificity, while another study [12], identified different species-specific mutations responsible for the *O* allele in human and primate species. Another species in which the *ABO* gene was described is the pig (*Sus scrofa*) [13].

The porcine *ABO* gene is located on *Sus scrofa* chromosome 1 (SSC1) at position 272898914–272935370 bp. Similarly to the human *ABO* gene, it is composed of eight exons, but only two alleles for the *N*-acetyl-galactosaminyl transferase gene have been observed: *A* and *O*. Allele *A* consists of the complete gene sequence, while allele *O* contains a large deletion of 2.3 kb encompassing the whole exon 8. This mutation determines a protein sequence ~60% shorter when compared to the 365 amino acids of allele *A*, with only one out of eight active sites being maintained. The deletion is caused by the intrachromosomal recombination between two Short Interspersed Nuclear Elements (SINEs) [14].

This deletion was hypothesized to be a trans-species polymorphism under balancing selection, originating more than 3.5 million years before the present (MYBP) in a common ancestor of the *Sus* species [14]. The hypothesis of balancing selection having a role in this *ABO* polymorphism has already been shown to be valid for primates, where alleles *A* and *B* were the result of a trans-species polymorphism that dated back more than 20 MYBP and is identical by descent in humans, gibbons, and Old World monkeys, while allele *O* appears to be species-specific [15].

The effect of the porcine alleles *A* and *O* on productive traits is not known yet, and neither is the presence or possible effects of other mutations of the gene in the variety of pig breeds and populations; the deletion that causes allele *O* was associated with lower levels of transcript (about 1/3 compared to that of allele *A*) and a decreased concentration of *N*-acetyl-galactosamine in the pig gut, leading to a lower abundance of *Erysipelotrichaceae* bacteria [14].

The aims of this study were (i) to obtain a comprehensive profile of *ABO* gene variants in *Sus scrofa* and related species that could shed additional light on the evolutionary history of this gene at the Suidae family level and (ii) to test the association of these variants with productive and blood-related traits in domestic pigs. To do so, we mined more than 500 genomes from different Suidae species, *Sus scrofa* populations, and breeds raised in different countries (derived from publicly available and de novo-produced sequencing data information) to describe the distribution of the *ABO* genotypes (determined by alleles *A* and *O*) and obtain a detailed map of genetic variants at this gene. Then, we used whole genome sequencing data obtained from Italian Large White pigs to run an explorative association between *ABO* gene variants and several phenotypes analyzed on the same animals.

## 2. Materials and Methods

### 2.1. Animals

The animals included in this work were not raised or treated in any way for the purpose of this study, and for this reason, no other ethical statement is needed.

This study included 529 whole-genome sequencing (WGS) datasets representing 1301 genomes related to 64 pig breeds and populations. Most data were publicly available from the European Nucleotide Archive (ENA) database (https://www.ebi.ac.uk/ena, accessed on 12 July 2023). Additional samples from three relevant Italian pig breeds were also included in this study. For most of the samples, one WGS dataset corresponded to genome sequences from one individual pig, whereas 23 WGS datasets derived from a DNA pooling strategy (that means more pigs per dataset). Datasets were organized as follows:

(i) ENA datasets (publicly available) were obtained from the sequencing of the genome of single animals [depth of sequencing (DP) > 5X over the whole genome and DP > 5X over the *ABO* gene and its 5 kb flanking regions]. This dataset was derived from 191 pigs from 20 European breeds, 105 pigs from 17 Asian breeds, 14 pigs from two breeds raised in South America, and two pig samples from one breed raised in New Zealand. Datasets from wild boars were also retrieved from the same database: two were from wild boars of the Near East region; 18 from wild boars of Asia; and 14 from wild boars of Europe. More details of this dataset and related sequencing-derived statistics are reported in Bovo et al. [16] and in Table S1;
(ii) ENA datasets (publicly available) were obtained from a DNA pooling strategy (DP > 20X). This dataset consisted of 22 DNA pools (constructed from 30–35 pigs each), representing 22 different European breeds and one European wild boar population. More details of this dataset and related sequencing-derived statistics are reported in Bovo et al. [17,18] and in Table S1;
(iii) Novel WGS datasets were produced from Italian Large White (no. = 90), Italian Duroc (no. = 35), and Italian Landrace (no. = 35) pigs. These pigs derived from triplets of siblings (two gilts and one castrated male) that were individually performance-tested at the central genetic station of the National Pig Breeders Association (ANAS) for the sib-testing evaluation of a boar from the same litter [19]. Animals were selected to be unrelated as much as possible based on pedigree analyses. Genomic DNA was extracted as described by Bovo et al. ([20]). Libraries with an average insert size of 350 bp were obtained and sequenced on the BGISeq 500 platform with a 150-bp long paired-end strategy, following the provider’s procedures. About 56 Gb were obtained from each pig, providing a DP of about 20X. More information on this dataset is reported in Table S1;
(iv) ENA datasets from additional *Sus* species (Table S1) were included in the analyses, as these species are phylogenetically close to *Sus scrofa* [21]. These data were from *Sus barbatus* (no. = 3), *Sus cebifrons* (no. = 9), *Sus celebensis* (no. = 2), and *Sus verrucosus* (no. = 3). Since all extant *Sus* species, except *Sus scrofa*, are present in the Island Southeast Asia (ISEA) [21], we indicate them as ISEA species. Moreover, to obtain more informative phylogenetic analyses, we also included WGS datasets from two non-*Sus* species of the *Suidae* family (Table S1): *Porcula salvania* (no. = 6); and *Phachochoerus africanus* (no. = 15).

### 2.2. Sequencing Data Analysis and Detection of the 2.3 kb Insertion Allele

To detect the presence of the 2.3 kb insertion and its zygosity state in each WGS dataset (and its allele frequency from the DNA pools), sequencing data were aligned against two pig reference genomes, as previously performed by Yang et al. ([14]): (i) the standard *Sus scrofa* reference genome (Sscrofa11.1; GCF_000003025.6); and (ii) a modified version of the *Sus scrofa* reference genome that included the 2.3 kb deleted sequence derived from the Berkshire genome (GCA_001700575.1). This latter version was named Sscrofa11.1+*ABO*. Mapping of the reads against the two reference versions was carried out with BWA 0.7.17 [22], using the *mem* function and applying default parameters. Then, duplicated reads were removed with Picard v.2.1.1 [23]. To obtain high-quality datasets and reliable detection of variants, reads with mapping quality Q < 50 were removed with Samtools 1.7 [24].

### 2.3. Sequencing Data Analysis and Detection of Other ABO Gene Region Variants

The genome region, including the *ABO* gene and its 5 kb flanking sequence on each side (SSC1:272,893,914–272,940,370 bp; 46,456 bp in total), which we will refer to as the expanded *ABO* gene region, was extracted and, for several animals, was visually inspected with IGV v.2.16.1 tool [25]. We then proceeded with detecting alleles *A* and *O* by characterizing each animal dataset with an approach exploiting the depth of sequencing (DP) of this region. For all datasets, the DP of each sequenced nucleotide of this region was obtained using Mosdepth v.0.3.3 [26]. According to the depth outputs, the following counts were retrieved:

(i) Average DP of the expanded *ABO* gene region (DP_ABO_); this count was computed for alignments over both versions of the reference genome (Sscrofa11.1 and Sscrofa11.1+*ABO*);
(ii) Average DP of the 39 bp sequence between the two deletion breakpoints (DP_BP_); this count was available only for the canonical Sscrofa11.1 reference genome;
(iii) Average DP of the 2.3 kb deleted sequence (DP_del_); this count was available only for the Sscrofa11.1+*ABO* genome version.

**Figure 1:**
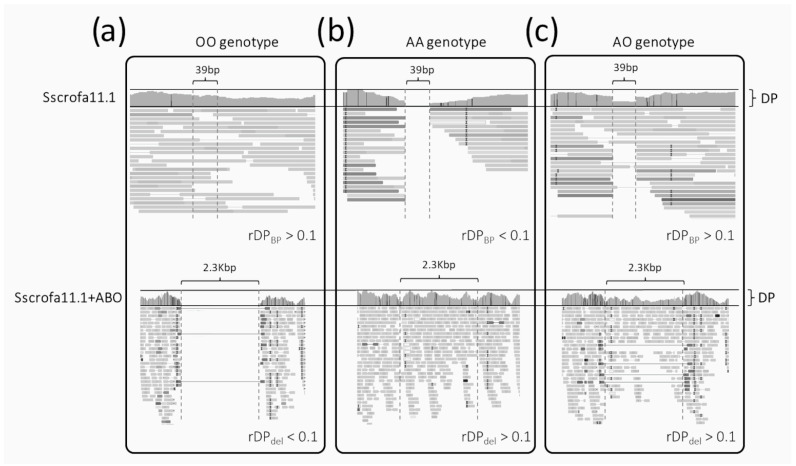
Depth of sequencing (DP) for the Sscrofa11.1 (top) and Sscrofa11.1+*ABO* (bottom) reference genomes for the three *A*/*O* genotypes: (**a**) *OO* genotype (homozygous genotype for the deletion of 2.3 kb); (**b**) *AA* genotype (homozygous genotype for the presence of the 2.3 kb region); and (**c**) *AO* genotype (heterozygous genotype).

These counts were also considered as ratios to derive two indexes that, when considered together, allowed us to correctly determine the *A*/*O* genotypes: $rDP_{del}=\frac{DP_{del}}{DP_{ABO}}$ and $rDP_{BP}=\frac{DP_{BP}}{DP_{ABO}}$. Based on these counts and indexes, alleles and genotypes of each animal sample were derived as follows:

(i) The homozygous *OO* genotype (i.e., deletion of the 2.3 kb sequence in both alleles; Figure 1a) is expected to have (i) reads covering the small region surrounded by the two breakpoints (DP_BP_ > 0) when investigating the alignment over the canonical Sscrofa11.1 genome and (ii) no reads mapped over the 2.3 kb sequences when analyzing the alignments over the Sscrofa11.1+*ABO* genome version (DP_del_~0). Considering ratios between counts and their simultaneous evaluation across the two reference genomes, genotype *OO* should have rDP_del_ < 0.1 and rDP_BP_ > 0.1;
(ii) The homozygous *AA* genotype (i.e., presence of the 2.3 kb sequence in both alleles; Figure 1b) is expected to have (i) no reads covering the small region surrounded by the two breakpoints (DP_BP_ < 0.1; theoretically zero) when investigating the alignment over the canonical Sscrofa11.1 genome and (ii) reads mapped over the 2.3 kb sequences (with a similar average DP of the whole gene) when analyzing the alignment over the Sscrofa11.1+*ABO* genome version (DP_del_ > 0). Considering ratios between counts, genotype *AA* should have rDP_del_ > 0.1 and rDP_BP_ < 0.1;
(iii) The heterozygous *AO* genotype (Figure 1c) is expected to have (i) half of the reads fully covering the small region surrounded by the two breakpoints (DP_BP_ > 0) and (ii) reads mapped over the 2.3 kb sequences (with half of the DP of the whole gene) when analyzing the alignment over the Sscrofa11.1+*ABO* genome version (DP_del_ > 0). Considering ratios between counts, the genotype *AO* should have rDP_del_ > 0.1 and rDP_BP_ > 0.1.

Figure S1 shows the relationship between the different DP counts and statistics when evaluating the three *ABO* gene genotypes. 

It is worth noticing that the reference genome (allele *O*) is characterized by the presence of a sequence of 287 nt derived from the intrachromosomal recombination of the duplicated SINE sequences (proximal and distal breakpoints of the 2.3 kb; see the Extended Data, Figure 5a, by Yang et al. [14]). This recombination event caused the fusion of the two SINE sequences into one, with the resulting SINE containing part of the left side SINE and of the right side SINE, joined by an 83 bp sequence, homologous between the two SINEs (from SSC1:g.272907337 to SSC1:g.272907419). From WGS datasets of animals carrying the 2.3 kb sequence, it would be expected to retrieve reads mapping up to position SSC1:g.272907419 from the left side and up to SSC1:g.272907337 from the right side of this shared 83 bp sequence before showing signs of soft clipping (i.e., portions of the read not aligning to the reference genome). We, however, observed that reads from animals carrying allele *A* mapped up to position SSC1:g.272907350 from the left side and up to position SSC1:g.272907389 from the right side (Figure 1b). This could be due to the level of homology between the two SINE sequences and the 83 bp sequence not being high enough and causing soft clipping of the reads earlier than expected. Thus, datasets putatively carrying the 2.3 kb deleted sequence were characterized by the presence of a 39 bp region surrounded by soft clipped reads and identifying two breakpoints (at position SSC1:272907350 and SSC1:272907389). When inspecting the same sequenced animal having the reads mapped over the Sscrofa11.1+*ABO* version of the genome, the full region was covered by reads, and no soft-clipped reads were detected, as expected.

For DNA-pool samples, the frequency of the deletion was estimated with rDP_del_ only.

### 2.4. Short and Structural Variant Discovery in the ABO Gene Region

The expanded *ABO* gene region was investigated for the presence of single nucleotide variants (SNVs, not including insertions/deletions, INDELs) and additional structural variants (SVs). 

Structural variants that could not be detected with common variant callers were identified with DELLY v1.1.6 [27]: SVs were jointly called and then filtered with the default DELLY parameters for germline variants. To check the origin of these SVs (i.e., repeats and low-complexity DNA sequences), genome annotation produced by the RepeatMasker tool [28] was used. For each SV, only the repeat with the highest coverage of the SV region was retained. 

Single nucleotide variants were called across all WGS datasets (jointly; also including the non-*Sus* species), using BCFtools v.1.16 [24], considering the alignments against the Sscrofa11.1 reference genome. Single nucleotide variants were then filtered by retaining only biallelic variants with (i) quality (Q) > 20, DP (at the single WGS dataset) between 5 and 70, and (ii) call rate > 0.9. SNVs were then annotated with the Ensembl Variant Effect Predictor (VEP) tool [29]. Single nucleotide variants were then used in a Principal Component Analysis (PCA), as implemented in PLINK v1.9 [30]. 

Linkage disequilibrium (LD) was computed for SVs and SNVs separately and visualized with the program LDBlockShow v1.40 [31]. For SNVs, only variants with minor allele frequency (MAF) > 0.05 were included in the LD analysis. Then, blocks of LD were identified based on SNVs having a *r*^2^ > 0.9 and the ratio of strong LD SNVs in one block higher than 0.9.

### 2.5. Phylogenetic Analysis and Introgression Evaluation

A phylogenetic tree was computed based on the genome sequence spanning the region SSC1:272,906,775–272,907,350 bp, which corresponds to the genomic positions of the end of the SVs having the highest LD value with the 2.3 kb deletion and the start of the 2.3 kb deletion (575 bp). Sequences, including the alternative allele for each SNV present in the region, were retrieved with the BCFtools consensus tool [24] for only those WGS having the *AA* or *OO* genotypes. Sequences having 100% sequence identity were collapsed under a single identifier and defined as a cluster. A multiple sequence alignment was built with MUSCLE [32], and a maximum likelihood tree was computed using IQTREE v2.2.2.3 [33]. ModelFinder software v2.2.2.3 [34] was used to select the optimal substitution model and was run with 2000 cycles of Ultrafast Bootstrap Approximation with UFBoot to assess branch support.

For the study of introgression, SNVs were initially phased using Beagle v.5.4 [35]. The porcine genome recombination map required by Beagle was obtained from the study of Tortereau et al. [36]; as this map was based on the previous version of the reference genome (Sscrofa10.2), genomic coordinates were updated to the Sscrofa11.1 using the UCSC liftover utility (accessed on 18 July 2023) [37]. Segments of identity by descent (IBD) origin were identified as a proxy of introgression relying on phased SNVs and the RefinedIBD tool [38]. Assessment of possible introgressed segments was based on the LOD score and length of the segments. With this approach, we tested all possible introgression events, both among species and among breeds and populations.

As a second approach to measure introgression, D-statistic (also known as ABBA BABA statistics) was computed only among species with the tool Dsuite v.0.5 [39]. *Phachochoerus africanus* was considered the outgroup species, as this is one of the parameters required by the tool. Trios of species were tested for introgression, and the Z-score of the D-statistic was used as a measure of significance. A Z-score > |3| was used to claim introgression [40].

### 2.6. Association Analyses with Production Traits and Blood Parameters in Italian Large White Pigs

For 90 sequenced Italian Large White pigs, data on six production traits and 33 blood parameters traits were available. Production traits included average daily gain (ADG), lean cut percentage (LC), ham weight loss at first salting (HWLFS), ham weight (HW), backfat thickness (BFT), and feed conversion ratio (FCR). For these traits, random residuals were calculated as a measure that resulted from linear models that included sex, batch, date of slaughtering, age at slaughtering, and inbreeding coefficient as fixed effects. Details of trait evaluation and data processing and modeling are reported in previous studies [19,41].

The 33 blood-related traits included 15 hematological parameters (seven erythrocyte-related traits, six leukocyte-related traits, and two platelet-related traits) and 18 clinical-biochemical parameters (electrolytes and lipid-related, metabolism-related, and protein-related parameters). Here, residuals were calculated as measures of the traits and resulted from linear models that included the date of slaughtering and sex and weight of the animals as fixed effects. Details of trait evaluation and data processing and modeling are reported in Bovo et al. [42,43].

Association analyses between *ABO* gene variants (SNVs and SVs, including the 2.3 kb deletion) and the traits described above were carried out using Linear Mixed Models. Additive genetic models assuming a trend per copy of the minor allele were used to specify the dependency of each trait on genotype categories. All models were fitted using GEMMA v.0.98 [44] after computing and including in the models a centered genomic matrix (K). To avoid underestimation of the stratification due to the short genome fragment analyzed and to account for the relatedness between the pigs analyzed, the relatedness matrix was computed based on genotypes obtained from the Illumina PorcineSNP60 BeadChip v.2 (Illumina Inc., San Diego, CA, USA), which was used on the same pigs. For each trait, each genetic marker was defined as associated based on the following two thresholds defined by the Wellcome Trust Case Control Consortium [45] and by several other studies in livestock (e.g., [46,47]): *p* < 5.00 × 10^−5^ was considered as significant, and *p* < 5.00 × 10^−4^ was considered as suggestively significant.

## 3. Results

This study focused on the *ABO* gene region and mined a total of 529 high-quality WGS datasets, covering 64 *Sus scrofa* breeds and populations, 17 datasets representing additional four *Sus* species, and 21 other datasets representing two other species of the *Suidae* family. The average depth of sequencing (DP) for the *Sus scrofa* datasets retrieved from ENA was 13 ± 4X, whereas the average DP of the *ABO* extended gene region was similar and equal to 11 ± 4X. The Italian Large White, Italian Duroc, and Italian Landrace sequenced pigs had an average DP of 23 ± 1X and 23 ± 2X for the gene region. Pooled WGS datasets had an average DP of 41 ± 6X and 39 ± 6X for the gene region. Datasets not produced from *Sus scrofa* had an overall average DP of 16 ± 7X and 15 ± 8X for the *ABO* gene region (see Table S1 for individual sequencing statistics).

### 3.1. A/O In Silico Genotyping Results

Among the 544 datasets derived from WGS of single animals, 22 breeds or species had a number of sequenced animals of five or larger (Table 1) and were considered acceptable for the first estimation of the *A*/*O* genotypes and allele frequencies. These datasets represented 19 pig breeds (seven Asian breeds, 11 European breeds, and one breed from South America) and three members of the *Suidae* family (*Phacochoerus africanus*, *Porcula salvania*, and *Sus cebifrons*). Allele *O* was observed in all 19 *Sus scrofa* breeds, with a minimum frequency of 0.31 in the Göttingen Minipig and up to being fixed with a frequency of 1.00 in the Korean pig (Table 1). Allele *O* was also identified in 24 out of 27 breeds with a sample size lower than five (Table S1). Allele *O* was also detected in the ISEA species (*S. barbatus*, *S. cebifrons*, *S. celebensis*, and *S. verrucosus;* Table S1); allele frequencies were estimated only in *S. cebifrons* (0.78). Considering the non-*Sus* species of the *Suidae* family, the allele *O* was fixed both in *Phacochoerus africanus* and *Porcula salvania* (Table 1).

We then focused on WGS datasets obtained from a DNA-pool sequencing approach applied to some cosmopolitan and autochthonous European pig breeds and wild boars. As for the previously described samples, allele *O* was present in all these datasets (Table 2); its frequency ranged from 0.29 (Mora Romagnola) to 0.93 (Italian Landrace). As for some populations/breeds (European Wild Boar, Italian Duroc, Italian Landrace, Italian Large White, and Swallow-Bellied Mangalitsa), the results were obtained both from DNA pools of individually sequenced animals, and the estimated frequencies derived from the two approaches were compared: there was a complete agreement on the allele frequencies (Pearson’s correlation > 0.99; *p* < 0.01), suggesting that the applied methods and sequenced pigs might provide unbiased information on the allele distribution at this gene.

### 3.2. Other Structural Variants and Linkage Disequilibrium Study in the ABO Gene Region

**Figure 2:**
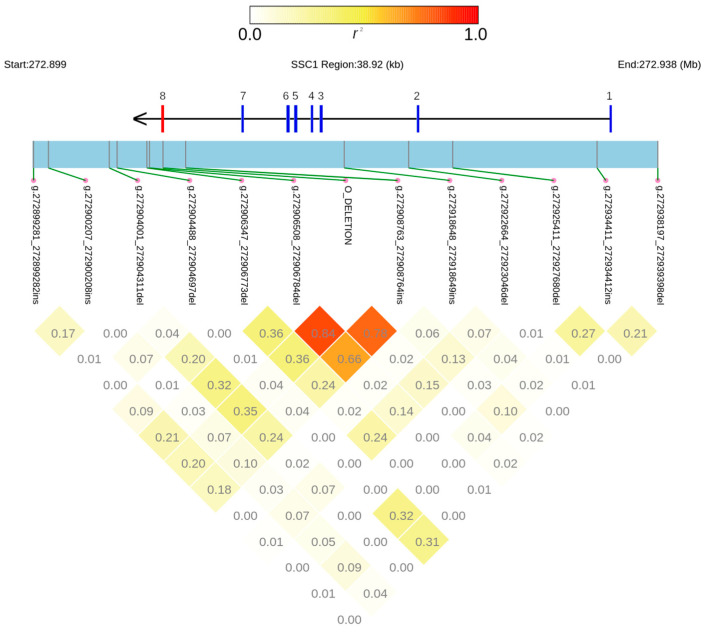
Linkage disequilibrium plot of structural variants, including the *A*/*O* polymorphic site at the *ABO* gene for all analyzed datasets. Numbers in the squares correspond to the *r*^2^ value; colors of the squares range from white to red, following the value of *r*^2^. Structural variants are reported along the gene structure, which includes the position of the deleted 8th exon (red).

A total of 12 other SVs were identified across all samples (Table S2), five insertions (ranging in size from 26 to 46 bp) and seven deletions (from 209 to 2269 bp). According to the VEP analysis based on the NCBI genome annotation (GFF file of Sscrofa11.1), six of them were located in intergenic regions; four were in intronic regions; one was downstream of the gene, and one was upstream of the gene (Table S2 and Figure 2). However, considering that the NCBI annotation lacks the eighth exon, the six intergenic variants should be considered as downstream variants with respect to the complete gene (i.e., allele *A*). For the same reason, the insertion g.272908763_272908764ins, annotated as a downstream gene variant on the reference genome, should be considered as an intronic variant with respect to the complete sequence of the *ABO* gene.

Eleven of these SVs overlapped with at least one of the repeats previously identified and available as a masked file in NCBI. For completeness, the 2.3 kb deletion was also included in this analysis despite being not detected with DELLY (Table S2). Seven SVs overlapped with the Pre0_SS SINE, including the deletion of allele *O*, one with the SINE1C_SS, two with the Long Interspersed Nuclear Elements (LINEs) L1MB5 and L1B_SS, and two with the Long Terminal Repeats (LTR) LTR10B2_SS and LTR39_SSc. The 83 bp region containing the deletion breakpoints identified for the deletion of allele *O* was found to be part of a SINE of 138 bp; this provided additional confirmation that the two SINEs in allele *A* combined into a single SINE in allele *O*. Among the detected SVs, only the SSC1:g.272908763_272908764ins was present and segregated in all species. It was worth noting that SSC1:g.272906508_272906784del was present in all species except in *Phachochoerus africanus* (Table S2).

Linkage disequilibrium analysis based on all *Suidae* datasets showed a high *r*^2^ value (*r*^2^ = 0.84) between the novel SV SSC1:g.272906508_272906784del and the 2.3 kb deletion, characterizing allele *O*, indicating a non-random combination of the two deletions (Figure 2).

In particular, animals having the 2.3 kb deletion did not have the SSC1:g.272906508_272906784del. Excluding the *Phachochoerus africanus* (where this SV was not present), all other ISEA species had an *r*^2^ = 1 (perfect linkage), whereas in the *S. scrofa*, *r*^2^ was equal to 0.95. However, we cannot exclude that *r*^2^ = 1 was due to the low sample size of the ISEA species, whereas, for *Sus scrofa* samples, some possible errors in the estimation of the genotypes could be the reason for the absence of the perfect LD.

Another SV, the SSC1:g.272908763_272908764ins, appeared fixed in all species except *S. scrofa*. Linkage disequilibrium analysis showed a high *r*^2^ value (0.84) with the 2.3 kb deletion. The fact that this SV was fixed in all species, with the exception of *S. scrofa*, suggested that this SV could be caused by a deletion event that happened in *S. scrofa*. This SV overlapped with 210 bp of the Pre0_SS SINE.

### 3.3. ABO Gene Single Nucleotide Variants

**Figure 3:**
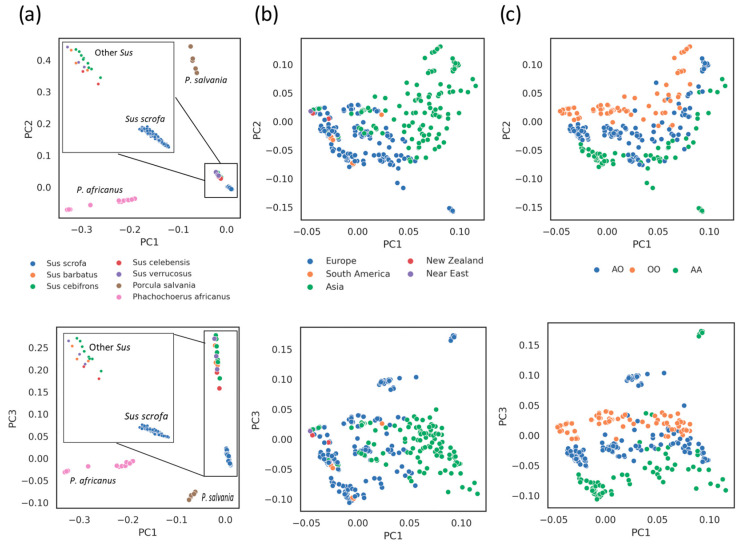
Principal Component Analysis based on Single Nucleotide Variants. The first three Principal Components (PC) are shown. Each dot represents a WGS dataset. (**a**) The analysis included all considered datasets from different species. Datasets are colored by species. (**b**) Analysis includes only *Sus scrofa* datasets. Datasets are colored by geographic origin of the breed/population. (**c**) Analysis includes only *Sus scrofa* datasets. Datasets are colored based on the *ABO* gene genotype (*AA*, *AO*, and *OO*).

The variant calling produced 4383 SNVs (Table S3), including 3210 (73%) private variants for the different species: 985 (22%) for *Sus scrofa*; 733 (16%) for the other *Sus* species; 371 (8%) for *Porcula salvania*; and 1121 (26%) for *Phachochoerus africanus.* A few variants (no. = 216, 5%) were segregated across all seven species investigated. Considering only *Sus scrofa* animals, 1965 (41%) variants were found in at least one pig sample, including 1187 SNVs (60%) already known and annotated in the Ensembl variation database (as accessed on August 2023). A Principal Component Analysis (component 1, PC1; component 2, PC2; component 3, PC3) based on these 4383 SNVs showed well-defined groups representing the different species included in the analysis (Figure 3a). Moreover, when considering the first three principal components of a PCA based on only *Sus scrofa* datasets and their SNVs (no. = 1965), a clusterization emerged, representing both the different breeds/populations (Figure 3b) and the three *ABO* gene genotypes, i.e., *AA*, *AO*, and *OO* (Figure 3c).

**Figure 4:**
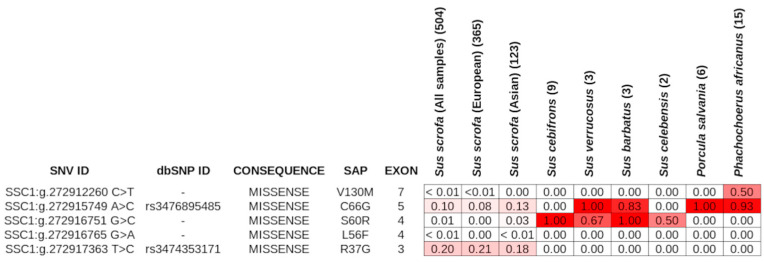
Missense variants (SNVs) identified in *Sus scrofa* datasets. The Single Amino acid Polymorphisms (SAP) and the frequency of the alternative allele across the different species are reported. The color of the cells ranges from white to red, and follows the allele frequency value. The number of animals included in the allele frequency estimation is reported within brackets in the correspondence of the analyzed species.

Among the detected SNVs in *Sus scrofa*, 12 variants were located in the exons of the gene. Five of these variants were missense mutations (Figure 4) and were located in the first 130 amino acids of the protein product.

None of them produced a single amino acid substitution affecting relevant protein sites, even when the substitutions were aligned against the *ABO* protein sequences of other mammals, including humans. However, the SSC1:g.272916751 G>C was responsible for the p.S60R, a protein substitution from serine, characterized by a polar uncharged side chain, to arginine, presenting a positively charged side chain. This variant was found to be polymorphic only in Asian pig breeds and ISEA species. Another SNV in exon 5 (SSC1:g.272915749 A>C) was found to be fixed in *Porcula salvania* and almost fixed in *Phacochoerus africanus*. Considering the different *Sus* species, (i) it resulted polymorphic in *Sus scrofa* and *Sus barbatus*, whereas (ii) *Sus cebifrons* and *Sus celebensis* did not have the alternative allele, and (iii) *Sus verrucosus* and *Sus barbatus* had a high frequency of the alternative allele (>0.8) (Figure 4).

Linkage disequilibrium analysis was performed considering the 916 SNVs (~21% of the identified SNVs) that had an MAF > 0.05 across all datasets (including all different species). Table S3 reports the LD blocks identified in this analysis. Across all samples, we identified a total of 28 LD blocks characterized by two or three contiguous SNVs in high LD (*r*^2^ > 0.9). However, considering that most of the SNVs were private for the different species, the LD was recalculated within each species. We focused mainly on *Sus scrofa*, as most of the information was available for this species. Here, 848 SNVs had MAF > 0.05 (~43% of SNVs present in at least one *Sus scrofa* sample). *Sus scrofa* samples contained 59 blocks with up to eight SNVs each, spanning genomic regions from 11 bp to 470 bp (60 bp on average). When considering their geographic origin, 730 SNVs and 1025 SNVs with MAF > 0.05 in European and Asian breeds, respectively, were retained. Here, we found 82 blocks in European breeds (from two to 14 SNVs each; covering up to 497 bp) and 50 blocks in Asian breeds (from two to six SNVs, covering up to 268 bp).

Linkage disequilibrium was then evaluated between SNVs and the 2.3 kb deletion (Table S4). Considering the pairwise LD between the *A*/*O* polymorphic site and the 916 SNVs with MAF > 0.05 identified across the different species, 16 SNVs had a value of *r*^2^ > 0.85. The highest LD value (*r*^2^ = 0.94) was found for the SNV SSC1:g.272909483 A>G (at a distance of about 2100 bp from the 2.3 kb deletion breakpoint). It is interesting to note that the SNV SSC1:g.272909483 A>G was fixed in *Porcula salvania* and *Phacochoerus africanus*, as well as the absence of the 2.3 kb deletion (indicating that allele *A* was fixed in these species). Within the *Sus scrofa* datasets (848 SNVs), 43 SNVs had a value of *r*^2^ > 0.85, whereas 29 had a *r*^2^ > 0.90. Considering European and Asian breeds separately, 61 and 10 SNVs had *r*^2^ > 0.90. It is worth noting that 10 variants identified in Asian breeds were also present in the European datasets. Of these 61 SNVs, eight were private to *S. scrofa*; 11 were found only in *Sus* species (absent in *Porcula salvania* and *Phacochoerus africanus*), and two were found in all species except *Phacochoerus africanus.*

### 3.4. Phylogenetic and Introgression Analyses

**Figure 5:**
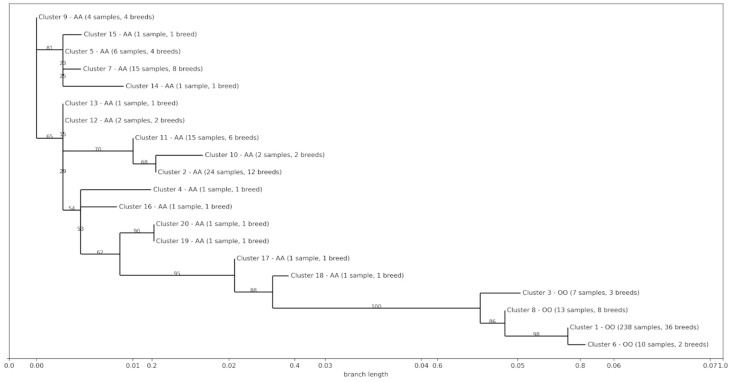
Maximum likelihood tree based on the SSC1:272,906,775–272,907,350 bp genome sequence (size = 575 bp) of animals carrying the OO and AA genotypes. Each node represents a cluster of sequences having 100% of sequence identity. For each node, the AA, AO, or OO genotype, the number of samples and breeds included in the cluster is also reported (details are reported in Table S7). The X-axis represents the branch length. For each branch, a branch support value is also reported in percentage.

The phylogenetic tree was computed for the ~600 bp sequence between the 2.3 kb deletion and SSC1:g.272906508_272906784del. This 600 bp sequence should be representative of the presence or absence of the 2.3 kb (due to LD). The phylogenetic tree was characterized by two main clusters (Figure 5), as also observed by Yang et al. [14], confirming that the in silico genotyping method implemented in our work worked properly.

No signature of introgression between species was found in the *ABO* gene region. The approach based on IBD did not return any genome segment positive for introgression, neither with default parameters nor with the lowest possible threshold acceptable by the tool. The same can be observed in the D-statistics, where no introgression events were retrieved.

### 3.5. Association with Productive Traits and Haematological Parameters

**Figure 6:**
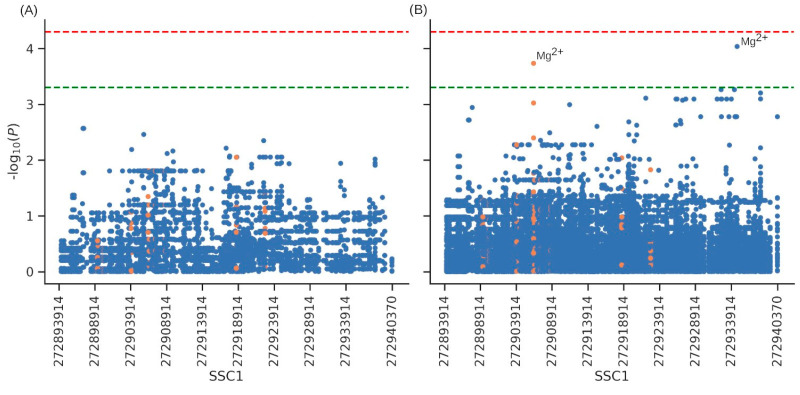
Over-imposed Manhattan plots focused on the *ABO* gene region, showing the results of the association analyses for (**A**) pig production traits and (**B**) the 33 blood-related parameters. Each dot represents either a single nucleotide variant (blue) or a structural variant (orange). The red and green lines identify significant and suggestive thresholds of association, respectively. Top associated markers are annotated with the name of the blood parameter.

Results of the association analyses for the six production traits are reported in Table S5. None of the SNVs characterizing the gene were associated with any of the six production traits considered here. The lowest *p* of association (*p* = 0.002) was detected for five SNVs with LC and ADG traits (Figure 6A and Table S5). The association analyses for the 32 blood-related parameters detected only one SNV in position 27,2934,734 bp (intronic) and one SV (SSC1:g.272906347_272906773del), both suggestively associated (*p* < 0.0001) with the level of the serum electrolyte Magnesium (Mg^2+^) (Figure 6B and Table S6).

## 4. Discussion

A detailed analysis of the genetic architecture of the *ABO* gene across species could provide important hints to explain the effect of variants on its functions and roles. Particularly, the investigation of this gene in pigs has multiple avenues of interest, as *S. scrofa* is a species of major economic relevance and is also considered an animal model with close similarities to humans, as well as one of the most promising sources for the development of xenotransplants [48]. Because pigs are primary candidates for organ donors for humans and are productive animals, the characterization of the porcine *ABO* gene has an important scientific relevance. The porcine *ABO* gene has only two major functional alleles, alleles *A* and *O*, and does not have a direct homolog of allele *B*, which has been reported in humans. This information is derived from previous studies [14,49] and can be confirmed by our study, which mined the genomes of more than 1300 pigs and other related species using a large number of WGS datasets.

In our study, both alleles *A* and *O* are present in pigs and ISEA species, with the exception of the Korean pigs, where allele *A* was not observed. *Porcula salvania* and *P. africanus*, distantly related to the *Sus* family, did not present the allele *O*.

We identified more than 4000 SNVs, including about 900 variants private for the *Sus scrofa:* SNVs distinctly group the different species and also different pig breeds. One SNV (SSC1:g.272909483 A>G) was found to be in high linkage disequilibrium with the porcine A/O polymorphic site across all different *Suidae* species and pig breeds and populations.

The analysis of structural variants along the *ABO* gene region detected 12 insertions and deletions, including a deletion (SSC1:g.272906508_272906784del) that was almost in perfect linkage with the *A*/*O* polymorphic position (and, thus, the two alleles) and is located at a distance of about 600 bp. This 275 bp deletion overlaps with a Pre0_SS SINE repeat. It is also worth mentioning that the expansion of Porcine Repeat Elements (PREs) is specific to the porcine lineage [50]. In our investigation, for at least 98% of the animals of the *Sus* lineages, this additional deletion was in LD with allele *A*, and it was never observed to be in linkage with allele *O*. The same was observed in the *Porcula salvania* datasets, which carried only allele *A* in complete LD with the SSC1:g.272906508_272906784del. The same haplotype structure was not observed in the *Phacochoerus africanus* sequenced animals, which carried only allele *A* without the SSC1:g.272906508_272906784del. A possible explanation for what we observed could be that allele O emerged after the divergence between *Phacochoerus africanus* and the other members of the *Suinae* subfamily, which occurred about 10 MYBP [21]. This event might have occurred in a haplotype that also included the SSC1:g.272906508_272906784del allele. Since none of the *Porcula salvania* animals carried allele *O*, it might also be argued that the deletion that caused this allele emerged after the divergence between the *Porcula* and the *Sus* genera (~3.5 MYBP; [21]). However, the presence of the additional deletion in all *Porcula salvania* samples indicates that this is not the case. To validate this hypothesis, more *Porcula salvania* animals should be sequenced to verify what could be observed from the six WGS datasets that we mined in our study. Another hypothesis is that *Porcula salvania* was obtained by interspecies introgression from the *Sus* species, the haplotype containing the additional deletion and allele A. Some evidence of interspecies introgression between *Sus scrofa* and *Porcula salvania* has already been reported [51]. To further test this hypothesis, we computationally evaluated introgression using Identity By Descent (IBD) analysis and D-statistics, including in the later test the information from *Phacochoerus africanus* as an outgroup. The choice of *Phacochoerus africanus* as an outgroup was motivated by the absence in all datasets of the haplotype, including allele *O* and the mentioned deletion, which strongly suggests that the *ABO* gene evolved independently in *Phacochoerus africanus* after its divergence from the *Suinae* sub-family lineage. It should, therefore, provide a good outgroup, being not so phylogenetically close to the *Sus* species and *Porcula salvania* but enough phylogenetically related to being informative. However, the results showed that no interspecies introgression events happened in that region.

Yang et al. [14] computed phylogenetic trees for the *ABO* gene in increasingly short windows around the *O* deletion. Their finding was that for shorter windows around the deletion, the *Phacochoerus africanus* allele *A* was closer to the *Sus* allele *A* than to the *Sus* allele *O*. Only one *Phacochoerus africanus* animal was investigated in their study. Their hypothesis was that allele *O* might, therefore, be older than the divergence between *Phacochoerus africanus* and the *Suinae* lineage. To test this hypothesis, we then computed a phylogenetic tree using the ~600 bp sequence contained between the 2.3 kb deletion and its linked SSC1:g.272906508_272906784del allele. The reason behind the selection of this polymorphic site was that the LD analysis confirmed that this region did not undergo any recombination, and, therefore, homozygous animals (*AA* and *OO*) can be used to construct the phylogenetic tree and observe the natural evolution that the sequences underwent the appearance of allele *O*. If allele *O* was exchanged between species through introgression, we would have expected a phylogenetic tree reflecting this state by placing the introgressed sequences and haplotypes close together. The results showed that this may not be the case as *Sus scrofa* sequences are clustered together, and no haplotypes from other species were found to be intermixed. When we considered *AA* and *OO* pig sequences separately, the *Suidae* lineage structure was reflected in the tree; possible exceptions were the *S. verrucosus* and *Porcula salvania AA* animals, which appeared to descend from a common ancestor, and the *Sus celebensis AA* animal, located closer to *Sus scrofa* sequences than to other ISEA animals. The hypothesis of the allele *O* being older than the Suidae lineage is contradicted by our findings, which show that none of the *P. africanus* samples present allele *O* nor the linked SSC1:g.272906508_272906784del, as described in the previous section.

The ABO group is not only expressed in erythrocytes but also in other tissues, with potential effects on several biological functions [1,14]. It is, therefore, important to investigate the association between polymorphisms in this gene and phenotypic traits in different pig breeds and populations. We, therefore, included an explorative association study between *ABO* gene polymorphisms and several production traits and blood-related parameters available in the sequenced Italian Large White pigs. The association analysis, which included about 600 variants, revealed no association with six production traits. This may be due to the relatively low number of animals included in this analysis, which might have affected the power of the association study for complex traits. A different scenario can be prospected when investigating internal phenotypes, such as blood-related and metabolomic traits, that normally need a lower number of samples in association analyses as few variants could explain a quite relevant fraction of the genetic variance of these phenotypes [52]. These traits are considered indicators of the physiological/health status of animals and might be indirectly related to economic traits [53,54,55]. In humans, *ABO* variants have been associated with hemoglobin levels, hematocrit traits, platelet count [8], and alkaline phosphatase levels [9]. In our explorative analyses carried out in Italian Large White pigs, one SNV and one SV have been suggestively associated with Mg^2+^ levels in serum. Magnesium is a fundamental component of meat quality, and dietary magnesium supplementation positively affects the behavior of animals, decreases their stress sensitivity, and improves pork quality by enhancing meat color, reducing drip loss, and increasing acidity [56,57]. None of these two genetic markers were in LD with the main 2.3 kb deletion, and this indicates that, potentially, other variations in the *ABO* gene may indirectly affect some phenotypes in pigs. These results suggest that *ABO* gene polymorphisms might not have any effect on routinely measured production traits in Italian Large White pigs. However, additional studies that include more animals, other breeds, and additional phenotypes are needed to obtain a better-defined picture of the effect of polymorphisms in this gene on economically relevant traits and other parameters that might be useful to understand the fine biological roles of the *ABO* gene in pigs.

## 5. Conclusions

The mining of hundreds of WGS datasets and genomes of different *Suidae* species, pig breeds, and populations made it possible to detect an *ABO* gene deletion not previously reported to be in high LD with the 2.3 kb deletion (allele *O*) that probably occurred between 3.5 and 10 MYBP. The allele distribution of these two polymorphic sites, together with several other SNVs and SVs, have been investigated in several tens of pig breeds, providing a general picture of variability in the *ABO* gene in many pig populations of different origins. In our explorative analysis, this novel deletion, together with the main *A*/*O* polymorphic site, were not associated with any production traits, but two variants were suggestively associated with Mg^2+^ hematological levels in Italian Large White pigs. Further studies are needed to verify the effect of the *ABO* gene in affecting microbiota and other traits and parameters in this breed and other breeds.

## Figures and Tables

**Table 1:** Genotype counts and allele frequencies of alleles *O* and *A* in breeds/species with at least 5 sequenced animals. Species, geographical origin, and number of analyzed samples are also reported.

Breed/Species	Origin	No. of Pig	Genotypes ^1^			Alleles ^2^	
			OO	AO	AA	O	A
*Sus scrofa* (total)		506	260	175	71	0.69	0.31
*Sus scrofa* (Asian only)							
Anqing Six-end-white pig	Asia	18	3	12	3	0.50	0.50
Asian wild boar	Asia	18	4	9	5	0.47	0.53
Erhualian	Asia	13	8	4	1	0.77	0.23
Jeju black pig	Asia	8	5	3	0	0.81	0.19
Korean pig	Asia	6	6	0	0	1.00	0.00
Meishan	Asia	34	14	15	5	0.63	0.37
Rongchang	Asia	5	1	2	2	0.40	0.60
*Sus scrofa* (European only)							
Duroc	Europe	36	11	18	7	0.56	0.44
European wild boar	Europe	14	1	5	8	0.25	0.75
Goettingen minipig	Europe	8	0	5	3	0.31	0.69
Italian Duroc	Europe	35	9	24	2	0.60	0.40
Italian Landrace	Europe	35	31	4	0	0.94	0.06
Italian Large White	Europe	90	67	20	3	0.86	0.14
Landrace	Europe	19	15	4	0	0.89	0.11
Large White	Europe	63	40	16	7	0.76	0.24
Mangalica	Europe	5	2	3	0	0.70	0.30
Pietrain	Europe	17	7	5	5	0.56	0.44
Yorkshire	Europe	18	4	6	8	0.39	0.61
Yucatan miniature pig	South America	13	4	7	2	0.58	0.42
*Sus cebifrons*	-	9	5	4	0	0.78	0.22
*Porcula salvania*	-	6	0	0	6	0.00	1.00
*Phachochoerus africanus*	-	15	0	0	15	0.00	1.00

^1^ No. of pigs with the indicated genotypes; ^2^ Allele frequencies.

**Table 2:** Allele frequencies at the *ABO* gene obtained from DNA pools for different European breeds and wild boars.

Breed/Population	Allele *O*	Allele *A*
Alentejana	0.51	0.49
Apulo Calabrese	0.72	0.28
Basque	0.47	0.53
Black Slavonian	0.53	0.47
Bísara	0.46	0.54
Casertana	0.52	0.48
Cinta Senese	0.85	0.15
European Wild Boar	0.35	0.65
Gascon	0.47	0.53
Italian Duroc	0.58	0.42
Italian Landrace	0.93	0.07
Italian Large White	0.80	0.20
Krškopolje	0.48	0.52
Lithuanian White Old type	0.84	0.16
Lithuanian Indigenous Wattle	0.77	0.23
Majorcan Black	0.37	0.63
Mora Romagnola	0.21	0.79
Moravka	0.69	0.31
Nero Siciliano	0.65	0.35
Sarda	0.51	0.49
Schwäbisch Hällisches Schwein	0.78	0.22
Swallow-Bellied Mangalitsa	0.29	0.71
Turopolje	0.61	0.39

## Data Availability

All publicly available data used for alignment are available via the European Nucleotide Archive (ENA; https://www.ebi.ac.uk/ena/browser/browser). Supplementary Table S1 reports sample and project identifiers for each evaluated dataset. In-house produced sequencing data related to the *ABO* gene region (Italian Large White, Italian Landrace, and Italian Duroc) can be shared after the signature of an agreement on their use with the University of Bologna. Please address all requests to luca.fontanesi@unibo.it.

## Supplementary Materials

The following supporting information can be downloaded at https://www.mdpi.com/article/10.3390/ani14010005/s1, Table S1: Whole-genome sequencing (WGS) datasets included in this study and in silico genotype of the alleles *A* and *O*; Table S2: Structural Variants (SV) identified in the expanded *ABO* gene regions. Information is based on the Sscrofa11.1 reference genome; Table S3: Single Nucleotide Variants (SNV) identified in the expanded *ABO* gene regions. Variant annotations, allele frequencies, and linkage disequilibrium blocks are provided; Table S4: Pairwise linkage disequilibrium (*r*^2^) of SNVs and the 2.3 kb deletion (allele *O*); Table S5: Results of the association analyses with production traits in Italian Large White pigs; Table S6: Results of the association analyses with blood-related parameters in Italian Large White pigs; Table S7: Information of the animals included in the nodes of the Maximum Likelihood tree; Figure S1: Relationships between the depth of sequencing (DP) counts and indexes in relation to the different *ABO* gene genotypes (*AA*, *AO*, and *OO*).
