# Supplementary material for: Investigation of ABO Gene Variants across More Than 60 Pig Breeds and Populations and Other Suidae Species Using Whole-Genome Sequencing Datasets
Source: Animals (Basel). 2023 Dec 19;14(1):5. doi: 10.3390/ani14010005 (PMC10778222; doi:10.3390/ani14010005)
Supplement: Supplementary file 1 [file animals-14-00005-s001.zip › animals-2688438-supplementary/Supp_Mat_ABO_animals REV v2.pdf]

Supplementary Materials

Investigation of *ABO* Gene Variants across More Than 60 Pig Breeds and Populations and Other *Suidae* Species Using Whole-Genome Sequencing Datasets

Matteo Bolner †, Francesca Bertolini *,†, Samuele Bovo †, Giuseppina Schiavo and Luca Fontanesi *

Department of Agricultural and Food Sciences, Division of Animal Sciences, University of Bologna, Viale Giuseppe Fanin 46, 40127 Bologna, Italy; matteo.bolner2@unibo.it (M.B.); samuele.bovo@unibo.it (S.B.); giuseppina.schiavo2@unibo.it (G.S.)

* Correspondence: francesca.bertolini3@unibo.it (F.B.); luca.fontanesi@unibo.it (L.F.)

† These authors contributed equally to this work.

Table S1: Whole genome sequencing (WGS) datasets included in the study and in silico genotype of the alleles *A* and *O*.

[data provided as a spreadsheet]

Table S2: Structural Variants (SV) identified in the expanded *ABO* gene regions. Information is based on Sscrofa11.1 reference genome.

[data provided as a spreadsheet]

Table S3: Single Nucleotide Variants (SNV) identified in the expanded *ABO* gene regions. Variant annotations, allele frequencies and linkage disequilibrium blocks are provided.

[data provided as a spreadsheet]

Table S4: Pairwise linkage disequilibrium (r^2) of SNVs and the 2.3 kb deletion (allele *O*)

[data provided as a spreadsheet]

Table S5: Results of the association analyses with production traits in Italian Large White pigs

[data provided as a spreadsheet]

Table S6: Results of the association analyses with blood-related parameters in Italian Large White pigs.

[data provided as a spreadsheet]

Table S7. Information of the animals included in the nodes of the Maximum Likelihood tree.

[data provided as a spreadsheet]

Figure S1. Relationships between the depth of sequencing (DP) counts and indexes in relation to the different *ABO* gene genotypes (*AA*, *AO* and *OO*).

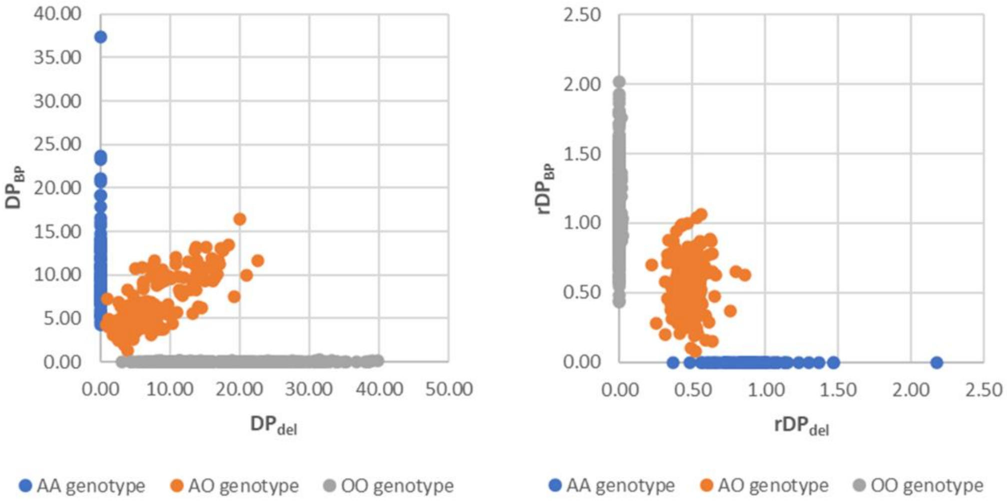

.
